# Information uncertainty: a correlate for acute stress disorder during the COVID-19 outbreak in China

**DOI:** 10.1186/s12889-020-09952-3

**Published:** 2020-12-07

**Authors:** Danhua Lin, Daniela B. Friedman, Shan Qiao, Cheuk Chi Tam, Xiaoyan Li, Xiaoming Li

**Affiliations:** 1 Faculty of Psychology, Beijing Normal University, Beijing, China; 2 Department of Health Promotion, Education, and Behavior, University of South Carolina, Columbia, SC USA; 3 South Carolina SmartState Center for Healthcare Quality, Arnold School of Public Health, University of South Carolina, 915 Greene Street, Room 408, Columbia, SC 29208 USA

**Keywords:** COVID-19, Information uncertainty, Acute stress disorder, China

## Abstract

**Background:**

Individuals’ stress in responding to the current COVID-19 pandemic may be exacerbated by information uncertainty driven by inconsistent, unverified, and conflicting news from various sources. The current study aims to test if information uncertainty during the COVID-19 outbreak was related to acute stress disorder (ASD) over and above other psychosocial stressors.

**Methods:**

An anonymous online survey was conducted with 7800 college students throughout China from January 31 through February 11, 2020. Existing scales were modified to measure ASD and six potential stressors including information uncertainty during the COVID-19 outbreak. Hierarchical regression analysis was conducted to assess the unique association of information uncertainty with ASD. To minimize the effect of large sample size and also to get a sense of whether the effects of information uncertainty were similar to people at the center of the epidemic, we repeated the hierarchical regression among 10% of the students who were randomly selected from the entire sample (“10% random sample”; *n* = 780) and 226 students from Hubei Province where the outbreak started.

**Results:**

Information uncertainty was highly prevalent among the respondents (64%). It was significantly associated with ASD beyond other key variables and potential stressors across three samples. In the hierarchical regression among the entire sample, demographic variables accounted for 9.4% of the variance in ASD. The other five stressors added 5.1% of the variance. The information uncertainty (β = .159; *p* < .001) explained an additional 2.1% of the variance. Likewise, the information uncertainty explained an additional 2.1 and 3.4% of the variance in ASD beyond all other variables among the 10% random sample (β = .165; *p* < .001) and the Hubei sample (β = .196; *p* < .01), respectively.

**Conclusion:**

Information uncertainty is a unique correlate of psychological stress during the COVID-19 outbreak. Reducing information uncertainty is essential not only for halting virus transmission but also for mitigating negative impacts of the pandemic on people’s psychosocial wellbeing. Transparent, timely, and accurate communication can reduce public confusion, fear, and stress. Capacity building in governments, communities, and media outlets to prevent, reduce and manage information uncertainty should be a critical part of the response to an emerging global health crisis such as the COVID-19 pandemic.

## Background

In early December 2019, a cluster of pneumonia-like cases were reported in Wuhan City of Hubei Province in central China [1]. Shortly after the onset of the epidemic, the disease was officially named as the Coronavirus Disease 2019 (COVID-19) by the World Health Organization (WHO) on February 11, 2020 and the severe acute respiratory syndrome coronavirus (SARS-CoV-2) was identified as the cause of the infection. On March 11, 2020, WHO declared COVID-19 a pandemic, pointing to the sustained risk of further global spread (https://www.who.int/news-room/detail/27-04-2020-who-timeline%2D%2D-covid-19).

Confronted with such a novel, highly contagious, and rapidly spreading virus, a need for compulsory isolation treatments and fears of cross-infection among a larger community and population prompted the Chinese government to lock down Wuhan City on January 23, 2020. It was the first time in Chinese history that a city with a population of more than 10 million was completely shut down. Many other cities in Hubei province as well as other provinces in China quickly followed and declared the first (and highest) level of public health response to the outbreak with a complete “lockdown” and nationwide travel bans, restricting all voluntary human movement to and from the cities by any means of public or private transportation. With the further outbreak of the infection, many cities in China have implemented similar controls on population movements by limiting voluntary traveling, public gathering and all entertainment and sports programs, and by delaying the normally scheduled reopening of all schools and factories after the traditional Chinese New Year holidays (which were initially scheduled to end on February 7, 2020). Many communities and neighborhoods also implemented “home-quarantines” where residents were typically permitted to leave their homes once or twice per week to purchase groceries or other essential items for living. Only those residents with valid identification, proof of residence, proof of being symptom-free, and an authorized pass were allowed to leave or enter their communities, neighborhoods, or villages. It was estimated that more than 150 million people were quarantined in China from late January through the middle of February 2020 [2]. In many regions of China, these mandatory measures have continued into the middle of March 2020. Both rapid spread of the virus and the accompanying drastic measures (e.g., nationwide lockdown) have resulted in multiple stressors such as the perceived severity of the infection and high infectability of the virus, interruption to their normal routine because of the lockdown, infection or suspected infection among family members or friends, family conflicts during the lockdown/quarantine, and many erroneous, inconsistent, unverified, and often conflicting news and messages (“information uncertainty”) during the outbreak [3, 4].

The unpredictable future of this pandemic has been exacerbated by information uncertainty, often driven by inconsistent, unverified, and sometimes conflicting news and messages from various governmental sources, social media outlets, and the Internet throughout China and abroad [5, 6]. Despite various measures taken by governmental and media sources to ease the concerns of communities, the lingering long-term outbreak, limited knowledge about this novel virus among both scientists and medical professionals, the inaccuracy of the information (e.g., origins of the virus, effectiveness of various control measures, number of people infected, the incubation period, and the mortality rate), the uncertainty about the pathogen and the transmission routes, are all contributing to intense stress among people living in China.

A vital aspect of crisis management and public health emergency response is the communication of timely and accurate information to the public, aimed to improve understanding of risks and to inform decision making [7]. Use of a primary source of information for the public during a disease outbreak can have great influence on perceptions of risk and people’s behaviors [8]. The information communicated can also influence perceptions of information certainty, self-efficacy, and intentions to follow guidelines from authoritative organizations [9]. Distrust of the messenger(s) or the sources of the information can affect people’s adoption of recommended behaviors especially if there exists uncertainty about the outbreak. As learned from other outbreak situations, clear, accurate, and timely communication to the public before and during an outbreak and clear inter-organization communication are critical [10]. While complete details about a public health crisis may not be known or available at the start of an outbreak, regular and cohesive communication from trusted leaders and organizations indicating that information is forthcoming is crucial. This is especially the case given the growing number of information outlets available to us, the potential for dissemination of misinformation by noncredible sources, and the rapid pace of messaging released to the public as experts gain a greater understanding about the outbreak [6]. This overload of quickly changing information, sometimes with questionable content and sources can be a contributor to the confusion, fear, and stress among the general population.

The rapid outbreak and spread of COVID-19 have affected many lives, created immense burden to healthcare systems, and resulted in huge economic losses on a global scale. Given the extent of questionable information that has emerged during this short period of time, we had a unique opportunity to examine the effects of information uncertainty on people’s psychological wellbeing during an outbreak. Therefore, we designed the current study to answer the following research questions: 1) Is information uncertainty prevalent during the outbreak? 2) Is information uncertainty a source of stress? 3) Is information uncertainty associated with acute stress disorder over and above other possible psychological stressors during the outbreak?

## Methods

### Design

An anonymous online survey was conducted among college students throughout China from January 31, 2020 (when the confirmed cases exceeded 10,000 in China) through February 11, 2020 (when the confirmed cases in China exceeded 50,000), which was considered as the peak of the COVID-19 outbreak in China [11]. The online survey was distributed using SO JUMP, a well-known Chinese Internet-based survey platform similar to Amazon Mechanical Turk [12].

### Participants

In order to recruit a relatively homogeneous sample in terms of their current schooling status, students were eligible to participate in the survey if they were currently enrolled in an academic program (bachelor, master, or doctoral) and were also willing to provide online consent. A total of 7941 individuals responded and consented to the survey. Data from 141 respondents were excluded because they were either not currently enrolled in universities (*n* = 67; e.g., reported as already graduated or prospective college students) or were determined to provide random or careless responses throughout the survey (*n* = 74), which leaves 7800 students in the current analysis.

### Procedure

After obtaining ethics approval from the Institutional Review Board of the Faculty of Psychology, Beijing Normal University (BNU), the online anonymous survey was distributed using SO JUMP. The online survey could be completed on multiple electronic devices, such as desktops, laptops, and smartphones. Students provided online informed consent and confirmed their voluntary participation prior to the survey. All invited participants were allowed to share the survey link with other college students. The SO JUMP survey system was set with an IP-based duplicate protection that allows only one submission per IP address. At the end of the survey, all participants were provided with a short section of guidance on psychological adjustment during the outbreak and also the phone number of a BNU-based hotline for free psychological counseling and support.

## Measures

### Demographics

The students were asked to provide brief demographic information including gender, age, year in college (e.g., freshman), major in college (e.g., engineering), and the province of their current residence (e.g., Hubei). For the purpose of data analysis in this study, the years in college were dichotomized into freshman/sophomore (1) vs. junior or higher (0). The majors in college were dichotomized into health-related (1) vs. non health-related (0). The students were also asked to rate their own health status along a 5-point scale ranging from “very poor” (1) to “very good” (5).

### Acute Stress Disorder (ASD)

Thirteen items from the ASD scale developed by Bryant and colleagues [13] were modified to assess ASD. The original ASD scale is a self-report inventory with 19 items that assess four groups of ASD symptoms (dissociative, reexperiencing, avoidance, and arousal). The sample items in the ASD scale included “feel numb or distant from your emotions” and “tried not to think about the outbreak”. The students were asked to indicate whether they had any of the listed symptoms in the past week with a 5-point response option (1 = “no symptoms” to 5= “very clear symptoms”). Because of the limited space in the online survey, we shortened the scale by randomly removing one or two items from each subscale (i.e., two items each from dissociative and avoidance symptoms and one item each from reexperiencing and arousal symptoms). A composite score was created by summing the responses to the 13 items with a higher score indicating a higher level of ASD. The Cronbach alpha for the 13 items was .94 for the current study sample.

### Psychological stressors

Nine items from the SARS-related Stressors Scale [14] were modified to assess the potential stressors during the outbreak. The nine items assessed a total of 6 potential stressors, including Stressor #1: *infection or suspected infection among relatives or friends* (two items; e.g., “relatives or friends were suspected or confirmed with coronavirus infection”); Stressor #2: *the interruptions, inconvenience, and chaos in their daily life* (two items; e.g., “schedules for daily life, work, or school have been interrupted and messed up”); Stressor #3: *information on the severity and high infectability of the virus* (one item; “heard or read discussions by others regarding the severity and high infectability of the novel coronavirus”); Stressor #4: *negative news from authorities* (one item; “heard or read many negative news from the authoritative channels regarding the epidemic”); Stressor #5: *family conflicts* (one item; “conflict with family members due to the epidemic”); and Stressor #6: *information uncertainty* (two items; e.g., “difficult to tell the authenticity of many online information regarding the epidemic”). Participants were asked whether they experienced any of these events (“*stressors*”) during the past 2 weeks with a dichotomous response (yes/no). For the stressors measured by two items, a composite dichotomous score was created by assigning a score of 1 if the response to one or both items was positive (yes) and a score of 0 if the responses to both items were negative (no). The main reason of such a dichotomization was to keep all the stressor measures on the same scale (yes/no).

### Data analysis

For the purpose of data analysis in this study, the ASD composite score was categorized into three levels by quantiles (bottom 25%, middle 50% and top 25%) in the bivariate analysis but used as a continuous variable in the multivariate analysis. Analysis of Variance (for continuous variables) or Chi-square (for categorical variables) was used to assess the bivariate differences of demographic characteristics by level of ASD. Hierarchical regression analysis was conducted to assess the effects of information uncertainty on the ASD taking into consideration of key demographic variables and other potential stressors. The continuous score of ASD was the dependent variable. All independent variables were entered subsequently into three regression models that were corresponding to three blocks of independent variables. The block 1 included only the demographic characteristics (i.e., gender, years in college, major in college, self-rated health status) that differed by ASD level in bivariate analysis. The block 2 included all the block 1 variables plus all the stressors except the information uncertainty. The block 3 contained all the block 2 variables plus the information uncertainty. To minimize the effect of a large sample size (“overpower”) and also to get a sense whether the effects of information uncertainty was similar to people at the center of the pandemic, we conducted the hierarchical regression analysis in three samples: entire sample (*n* = 7800), 10% of the students who were randomly selected from the entire sample via SPSS “Select Cases: Random Sample” procedure (“10% random sample”; *n* = 780), and 226 students from the Hubei Province where the outbreak started in China. Standardized beta (β) coefficient and its significance level were used to indicate the predictability of each independent variable. R-square (R^2^) and R-square change (ΔR^2^) were used to assess the contribution of each model in terms of the proportion of variance (R^2^) in ASD explained by each model and the differences (ΔR^2^) in such contributions between models. All statistical analyses were conducted using SPSS for Windows, version 26.0 (IBM Corp., Armonk, N.Y., USA).

## Results

### Sample characteristics

The participants (*n* = 7800) in the current study were from all 31 provinces, province-level municipalities, and autonomous regions in mainland China, including 226 students (2.9%) from Hubei Province, the center of COVID-19 outbreak in China. Among the participants, 38.5% of them were male and mean age was 20.54 years (*SD* = 2.11). The majority of the sample were undergraduate students (*n* = 7261; 93.1%), while the rest (*n* = 539; 6.9%) were graduate students (master or doctoral students). About one quarter (*n* = 1850; 23.7%) of participants majored in health-related disciplines (e.g., medicine, public health, nursing). About two-thirds of the participants (*n* = 6060; 77.7%) considered their health status as good or very good. One-fifth (*n* = 1578; 20.2%) considered their health status as “average” and a small number (*n* = 162, 2.1%) considered themselves having a poor or very poor health.

### Level of ASD and correlates

As shown in Table 1, self-rated health status was significantly associated with the level of ASD with poorer health status being associated with a higher level of ASD (*p* < .001). Gender was significantly associated with the level of ASD with more males (32.4%) than females (27%) reporting a low level of ASD. Years in college were also associated with ASD with more freshmen and sophomore (27.8%) than other students (26.3%) reporting a high level of ASD. More students with non-health-related majors (25.2%) than students in health-related majors (21.1%) reported a high level of ASD. The proportions of positive response to stressor measures ranged from .18 (*family conflict*) to .93 (*information on the severity and high infectability of the virus*). The information uncertainty was endorsed by 64% of the respondents. All six measures of potential stressors were positively associated with the level of ASD (all *p* < .001) with higher levels of ASD being associated with higher proportions of positive responses to the stressor measures.

**Table 1:** Sample characteristics by level of acute stress disorder (ASD) among Chinese students

N(%)	Overall	Level of ASD		
		Low	Medium	High
	7800 (100%)	2117 (27.1)	3792 (48.6)	1891 (24.2)
Age (Mean ± SD)	20.54 ± 2.11	20.60 ± 2.18	20.49 ± 2.09	20.56 ± 2.07
Health Status (Mean ± SD)	4.07 ± .81	4.44 ± .74	4.03 ± .74	3.74 ± .86***
**Gender**				
Male	3001 (38.5)	972 (32.4)	1273 (42.4)	756 (25.2)
Female	4799 (61.5)	1621 (27.1)	2519 (48.6)	1135 (24.2)***
**Years in College**				
Freshman and Sophomore	4216 (54.1)	1174 (27.8)	2072 (49.1)	970 (23.0)
Junior or Higher	3584 (45.9)	943 (26.3)	1720 (48.0)	921 (25.7)*
**Major**				
Health-related	1850 (23.7)	496 (26.8)	964 (52.1)	390 (21.1)
Non Health-related	5950 (76.3)	1621 (27.2)	2828 (47.5)	1510 (25.2)***
**Residence**				
Hubei Province	226 (2.9)	54 (23.9)	111 (49.1)	61 (27.0)
Non-Hubei Provinces	7574 (97.1)	2063 (27.2)	3681 (48.6)	1830 (24.2)
**Stressors**				
Stressor #1	.22 ± .41	.16 ± .37	.20 ± .40	.33 ± .47***
Stressor #2	.86 ± .35	.79 ± .41	.90 ± .29	.84 ± .36***
Stressor #3	.93 ± .26	.93 ± .25	.95 ± .21	.86 ± .35***
Stressor #4	.70 ± .46	.64 ± .48	.72 ± .45	.74 ± .44***
Stressor #5	.18 ± .38	.10 ± .30	.15 ± .36	.31 ± .46***
Stressor #6	.64 ± .48	.46 ± .50	.68 ± .47	.77 ± .42***

Note:

Stressor #1: Family or relatives are diagnosed or pending diagnosis

Stressor #2: Chaos in daily schedule due to the lockdown

Stressor #3: Information of the severity and high infectability of the virus

Stressor #4: Negative news from the authorities

Stressor #5: Family conflict caused by the epidemic/lockdown

Stressor #6: Uncertainty from various information about the virus or outbreak

*p < .05; *** *p* < .001

### Hierarchical regression analysis

#### Entire sample

As shown in Table 2, self-rated health status and major in college remained significant predictors to ASD across all three models for the entire sample (*n* = 7800). All five stressors in model 2 were also significantly predictive of ASD. When the information uncertainty was entered into the regression (model 3) as a significant predictor (β = .159; *p* < .001), one of the stressors (“*chaos in daily schedule*”) became non-significant. The block 1 variables (demographics) accounted for 9.4% of the variance in ASD (continuous variable). The other five stressors explained an additional 5.1% of the variance in ASD. The information uncertainty (block 3) explained an additional 2.1% of the variance in ASD beyond all other variables.

**Table 2:** Hierarchical regression analysis for acute stress disorder (ASD)

	Model 1 Standardized β	Model 2 Standardized β	Model 3 Standardized β
**Entire Sample (*n*** **= 7800)**			
Years in college (1 = Freshman)	.021	.023*	.023
Gender (1 = Male)	−.001	.003	−.004
Health	−.305***	−.270***	−.256***
Major (1 = Health-related)	−.050***	−.040***	−.037**
Stressor #1		.048***	.037**
Stressor #2		.056***	.010
Stressor #3		−.163***	−.166***
Stressor #4		.069****	.052***
Stressor #5		.120***	.099***
**Stressor #6**			.159***
R^2^	.094	.145	.166
ΔR^2^		.051	.021
**10% Random Sample (*n*** **= 780)**			
Years in college (1 = Freshman)	−.013	−.005	−.015
Gender (1 = Male)	.010	.043	.029
Health	−.236***	−.186***	−.181***
Major (1 = Health-related)	−.085*	−.068	−.065
Stressor #1		.113**	.100**
Stressor #2		−.003	−.053
Stressor #3		−.114**	−.118**
Stressor #4		.074*	.067
Stressor #5		.130***	.102**
**Stressor #6**			.165***
R^2^	.060	.120	.143
ΔR^2^		.060	.023
**Hubei Students (*n*** **= 226)**			
Years in college (1 = Freshman)	.086	.051	.071
Gender (1 = Male)	−.032	−.044	−.048
Health	−.253***	−.235***	−.217**
Major (1 = Health-related)	−.054	−.045	−.051
Stressor #1		.035	.016
Stressor #2		.051	.012
Stressor #3		−.219**	−.221**
Stressor #4		.166*	.145*
Stressor #5		.051	.020
**Stressor #6**			.196**
R^2^	.074	.130	.164
ΔR^2^		.056	.034

Note:

Stressor #1: Family or relatives are diagnosed or pending diagnosis

Stressor #2: Chaos in daily schedule due to the lockdown

Stressor #3: Information on the severity and high infectability of the virus

Stressor #4: Negative news from the authorities

Stressor #5: Family conflict caused by the epidemic/lockdown

Stressor #6: Uncertainty from various information about the virus or outbreak

*p < .05; **p < .01; *** *p* < .001

#### 10% random sample

The results of hierarchical regression analysis for the 10% random sample (*n* = 780) remained largely similar to the entire sample with a few exceptions. Major in college was no longer significant in models 2 and 3. Two of the stressors in Model 2 became non-significant (“*chaos in daily schedule*”, *“negative news from the authorities*”). However, information uncertainty remained significant in model 3 (β = .165, *p* < .001). The block 1 variables explained 6% of the variance in ASD; block 2 variables explained an additional 6% of the variance in ASD, while information uncertainty explained an additional 2.3% variance in ASD beyond the contributions of all other variables.

#### Hubei student sample

While a number of significant results were diminished (probably because of the relatively small sample size) in the regression analyses for the Hubei student sample (*n* = 226), the key findings remain similar. Self-rated health status remained a strong predictor for ASD in model 3 (β = −.217, *p* < .001). Two other stressors (“*information on the severity and high infectability of the virus”, “negative news from the authorities*”) remained significant in models 2 and 3. Information uncertainty remained a significant predictor for ASD in model 3 (β = .196, *p* < .01). The block 1 variables explained 7.4% of the variance in ASD; block 2 variables explained an additional 6.6% of the variance in ASD, while information uncertainty explained an additional 3.4% variance in ASD beyond the contributions of all other predictors.

## Discussion

Emerging public health threats such as the COVID-19 pandemic pose serious challenges in information and communication [15]. Information regarding the pandemic is often incomplete and conflicting, especially in the early stages of the crisis with a novel virus that is not fully understood [16]. The uncertainty and ambiguity of information including fake news, rumors, conspiracy theories, and pseudo-scientific claims may compromise the public’s response to the crisis and create mass confusion and anxiety [17]. The current study suggests that information uncertainty has been highly prevalent (64%) during the outbreak of the COVID-19 in China. Information uncertainty, as a source of stress, was significantly associated with ASD over and above other key variables and potential psychological stressors across all three samples. Our findings underscore the importance of rapid and efficient dissemination of accurate, credible, and verified information about the disease (transmission and consequences) and protective actions [18]. Our findings also support the findings from a recent review highlighting that adequate information is key in reducing the psychological impact of quarantine measures during the COVID-19 outbreaks [19].

It is also notable that hearing or reading about discussions of the severity and high infectability of the virus (Stressor #3) was not significantly associated with the elevation of ASD but actually served as a protective factor for ASD across all three samples in the current study. During a public health crisis, people naturally develop concerns about their own health and the health of their families. The accurate and timely information (even negative information) could help them assess their own risk and make appropriate decisions related to self-protection. Accurate and reliable knowledge about the virus might improve individuals’ self-efficacy to engage in preventive behaviors (e.g., washing hands regularly, wearing a face covering, following social distancing guidelines) and buffer their fears and anxiety. Our finding suggests that it is not the negative news but the “bad news” (unverified, inconsistent, and self-conflicting news) that causes stress. Concealing or withholding the negative news about the virus or epidemic to the public could be an ineffective or even detrimental strategy in crisis management which has been a key lesson learned from many countries during the early stages of the COVID-19 outbreak [20]. Reducing information uncertainty is essential not only for halting disease transmission but also for mitigating negative impacts of the pandemic on people’s psychosocial wellbeing [19]. Transparent and timely communication rather than ambiguous and conflicting information can reduce public confusion and stress and increase communities’ capacity to partner on prevention and risk mitigation strategies [21].

The current study also revealed several other psychosocial factors or stressors that may influence the ASD levels among the participants. For example, we found strong bivariate and multivariate associations between self-rated health status and ASD level with poor health status relating to high a ASD level, which is reasonable given that the individuals in poor health may be particularly vulnerable to the virus and mortality from the infection. These individuals may become more anxious and stressed compared with those in good health during such an outbreak. It is also interesting that the significance of “family conflicts caused by lockdown” (Stressor #5) with ASD diminished in the Hubei sample. Although this result may be induced by the small sample size (*n* = 226), it could be explained by the specific social context of Hubei Province during the COVID-19 pandemic. As the origin and epicenter of the COVID-19 outbreak, Hubei reported 67,666 confirmed cases with 2959 deaths by March 6, 2020 [22]. Hundreds of thousands of households in Hubei have endured a much higher infection and mortality rate from the coronavirus. Facing the huge threat of losing loved ones, family members may have become more harmonious than before or given much less weight to any conflicts that were caused by the outbreak or accompanying measures during the crisis.

The results in the current study need to be interpreted carefully due to several limitations. First, the participants in the study were college students. They were young adults with a high education level who had not yet entered the job market and might be generally less concerned with the condition of their health. Some of them might worry about the interruption of their plans for graduation and job seeking but generally they might not have a heavy financial burden or real life stress. Some measures of psychological stressors, such as interruption of daily life, might have less of an impact on college students than their parents and older age groups. Therefore, their ASD level may not be representative of other populations. In addition, compared to other populations with less education, college students might face less information uncertainty as they can distinguish between fact versus fiction better. However, college students constitute an appropriate population for studing the effects of information uncertainty as they may be more familiar with and more connected to social media, and therefore have a higher chance of being exposed to conflicting information and information uncertainty compared to other populations (i.e. elderly, children). Second, the participants were recruited online through convenience sampling. The sample may be subject to voluntary bias and is not representative of all college students in China. Third, the survey questionnaire used in the study was short because of the logistic and feasibility concerns of an online survey. Therefore, data were not available on some important factors (e.g., the types and sources of uncertain information) and many other sociodemographic variables (e.g., pre-existing mental health conditions, family socioeconomic status) that may confound the relationship between information uncertainty and ASD. In addition, the ASD scale was shortened for the same reason and such shortening may result in an underestimation of ASD symptoms among the participants. It is also possible that the shortening of the original scale by randomly removing scale items may negatively impact the reliability and validity of the scale. However, such a negative effect was not evidenced from the results of the current study. The Cronbach alpha for the shortened scale (e.g., 13 items) in the current study was .94 which was similar to the Cronbach alpha (.90) reported for the original 19-item scale [13]. The comprehensive validation of the shortened scale is beyond the scope of the current study. However, the results in the current study supported a strong validity as the ASD scale was significantly associated with all six stressors as we hypothesized.

Despite these limitations, the current study is one of the first efforts to explore the influence of information uncertainty on people’s psychosocial wellbeing in responding to this emerging public health crisis. The prevalence of information uncertainty indicates the gaps in information and communication management during the epidemic. How to convey accurate information in a timely manner to the public is an immense challenge for governments worldwide. Experiences in coping with global public health crises such as H1N1 influenza, Ebola, and Zika have shed insights on strategies for reducing and managing information uncertainty. For example, during the Ebola outbreak in 2014–2015, federal and state guidelines for managing the potential community spread of Ebola within the United States did not exist and news media coverage about major response policies such as risk-based restrictions were quite limited compared with topics such as quarantine and isolation, which were smaller components of the policies used to control the spread of disease [23]. Through this form of agenda setting, media coverage of pandemics can shape what the public ends up deeming to be important. The Crisis and Emergency Risk Communication (CERC) framework developed by the U.S. Centers for Disease Control and Prevention is guided by several theories including social marketing, diffusion of innovations, sensemaking, and cognitive learning, and is a relevant tool used to prepare public health professionals for communicating this type of information to the public [24]. Some studies encourage public information officers to play a more active role in social media monitoring and countering misinformation [25, 26]. Dredze and colleagues [27] noted that “public health officials must get out in front of the conspiracy theorists to educate and influence the population” (p.3442). Although information uncertainty seems to be unavoidable during the early stages of a new global health crisis (such as the COVID-19), optimal channels and strategies to effectively communicate essential information related to such a crisis or the virus warrant further research so that society can be better prepared to effectively manage information uncertainty and mitigate its negative impacts. The data in the current study have strongly suggested needs of training, capacity building, and coordination among leaders and organizations sharing critical messages to prevent, reduce, and manage information uncertainty in the response to an emerging global public health crisis such as the COVID-19 pandemic.

## Conclusions

Information uncertainty is a unique and significant correlate of psychological stress during a global health crisis such as COVID-19. It is not the negative news but the unverified, inconsistent, and self-conflicting news that may cause stress. Reducing information uncertainty is essential not only for halting virus transmission but also for mitigating the negative impacts of the pandemic on people’s psychosocial wellbeing. Transparent, timely, and accurate communication can reduce public confusion, fear, and stress and encourage engagement in preventive behaviors. Capacity building at various levels (government, community, media) to prevent, reduce, and manage information uncertainty should be a critical part of the response to an emerging global health crisis such as the COVID-19 pandemic.

## Data Availability

The datasets used and/or analyzed during the current study are available from the corresponding author on reasonable request.

## Abbreviations

ASD: Acute Stress Disorder
BNU: Beijing Normal University
